# Assessing the prognosis mortality in patients with cutaneous verrucous carcinoma using Lasso-cox regression model: a retrospective study

**DOI:** 10.1007/s12672-025-02893-6

**Published:** 2025-06-13

**Authors:** Santosh Chokkakula, Siomui Chong, Yu-Yen Yang, Liying Huang, Yanan Qian, Qixiang Sun, Sijian Xia, Xiaoxi Zhang, Jiang Yong, Balaji Pathakumari, D. S. Prabakaran, In-Cheong Si, Yuyi Ou, Chengliang Yin

**Affiliations:** 1 Department of Microbiology, Chungbuk National University College of Medicine and Medical Research Institute Cheongju, Chungbuk, 28644 South Korea; 2 Department of Dermatology, The University of Hong Kong-Shenzhen Hospital, Shenzhen, 518053 China; 3 Department of Dermatology, The First Affiliated Hospital of Jinan University & Jinan University Institute of Dermatology, Guangzhou, 510630 China; 4 Dr. Pong Dermatologic and Aesthetic Clinic, Taipei, Taiwan China; 5 Department of Clinical Research, The First Affiliated Hospital of Jinan University, Guangzhou, China; 6 Orthopedic Medicine Faculty, Henan University of Chinese Medicine, Zhengzhou, Henan China; 7 School of Radiology, Shandong First Medical University, Taian, 271016 Shandong China; 8 School of Basic Medical Sciences, Capital Medical University, Beijing, 100069 China; 9 Department of Oncology, The University of Hong Kong-Shenzhen Hospital, Shenzhen, 518053 China; 10 Division of Pulmonary and Critical Care Medicine, Department of Medicine, Mayo Clinic, Rochester, MN 55905 USA; 11 Department of Biotechnology, School of Bio-Engineering, SRM Institute of Science and Technology, Chennai, Tamil Nadu 603 203 India; 12 Department of Orthopedic, CLINICA DE WONG’S, Macau, China; 13 Department of Gynaecology, Foshan Women and Children Hospital Affiliated to Guangdong Medical University, Foshan, 528000 China; 14 Medical Innovation Research Department, Chinese PLA General Hospital, Beijing, 100853 China

**Keywords:** Cutaneous verrucous carcinoma, LASSO, Cox proportional hazard, Overall survival, Prognosis prediction

## Abstract

Elucidating risk factors and prognostic indicators for cutaneous verrucous carcinoma (CVC) is crucial for rapid medical intervention. This study examined CVC incidence risk and prognostic factors, emphasizing sex disparities. Utilizing SEER Database records, we analyzed patients diagnosed with primary CVC from 2004 to 2015. Multivariate logistic regression identified risk factors for the incidence model, while multivariate Cox regression developed the mortality prognosis model. Lasso regression and lasso Cox models determined key factors for respective models. Restricted cubic spline (RCS) models measured age-related risk associated with CVC presence and survival. The study included 1,125 CVC patients (668 males, 59.4%; 457 females, 40.6%) at the time of diagnosis. Lasso regression identified independent risk factors including age, sex, race, marital status, AJCC Stage, Combined Summary Stage, radiation, surgery, tumor size, chemotherapy, and regional lymph node involvement. The Age, sex, marital status, AJCC stage, combined summary stage, and surgery were independently associated with overall survival (OS) and statistically significant. Kaplan-Meier analysis revealed significantly shorter OS in female CVC patients compared to males (*P* < 0.05). The RCS model demonstrated a U-shaped pattern, indicating a significant nonlinear relationship between age and CVC incidence. The current study uncovered sex-related variations in incident risk and mortality prognostic factors, prediction of complications among CVC patients, offering valued insights for healthcare professionals in clinical assessments and interventions.

**Supplementary Information:**

The online version contains supplementary material available at 10.1007/s12672-025-02893-6.

## Introduction

Cutaneous verrucous carcinoma (CVC) represents a rare variant of well-differentiated squamous cell carcinoma (cSCC), characterized by indolent growth and low metastatic potential [1]. This neoplasm predominantly affects elderly Caucasian males, typically in the sixth to seventh decades of life [2]. Epidemiological data indicate a substantial increase in cSCC incidence, with reports suggesting a 50–200% rise over the past three decades [3]. Current estimates in the United States indicate approximately 1.8 million new cases annually [4]. Differential diagnosis of CVC poses significant challenges, particularly in distinguishing it from giant condyloma acuminatum (GCA) and pseudoepitheliomatous hyperplasia (PEH). This diagnostic complexity can result in delayed identification and subsequent treatment complications [5]. Despite extensive research on immunohistochemical markers for verrucous carcinoma, validated and reliable indicators remain elusive [6]. Consequently, CVC diagnosis continues to rely heavily on clinical presentation and histopathological features [7]. The paucity of reliable molecular biomarkers and the overlapping histological characteristics with other verrucous lesions underscore the need for improved diagnostic methodologies. This diagnostic uncertainty highlights the importance of comprehensive clinico-pathological correlation in the management of suspected CVC cases.

Several prognostic factors have been identified as high-risk indicators for CVC, including tumor size, anatomical location, recurrence, prior radiotherapy exposure, immunosuppression, and perineural invasion. Advanced-stage recurrent CVC is associated with a poor prognosis, characterized by an increased likelihood of parotid involvement, nodal metastasis, and suboptimal local-regional control. Despite aggressive surgical interventions, such as parotidectomy and neck dissection followed by adjuvant radiotherapy, five-year disease-free survival rates remain below 50%. Histopathological and clinical features play crucial roles in determining patient outcomes. Poorly differentiated carcinoma has been identified as an independent predictor of reduced disease-free survival, while advanced age independently correlates with diminished overall survival among various tumor characteristics. Interestingly, in cases involving parotid and neck involvement, no statistically significant differences were observed in disease-free or disease-specific survival rates. Furthermore, the specific location of nodal involvement did not significantly impact survival outcomes in patients with metastatic CVC [8, 9].

Addressing the clinical challenges posed by CVC necessitates the identification and evaluation of risk and prognostic factors in patients. Understanding the elements associated with disease progression, recurrence, and overall survival is essential for customizing treatment strategies and providing precise prognostic information. In recent years, the advent of medical big data and the application of machine learning have shown promise in efficiently processing large datasets. Researchers have developed various clinical models leveraging this combination, notably risk models, which serve as practical tools for clinicians to differentiate risk groups and tailor treatment plans accordingly. Consequently, several models, including the LASSO-Cox regression, have been established to evaluate the incidence and prognosis risk factors in carcinoma patients [9–13]. Least absolute shrinkage and selection operator (LASSO) regression, introduced by Tibshirani et al. in 1997, is a method for variable selection and shrinkage within the Cox proportional hazards model, utilizing a penalty function to refine the model further [14].

The LASSO-Cox model has demonstrated significant utility in cancer research across various malignancies, including lung adenocarcinoma, bladder cancer, gastric cancer, pancreatic cancer, and hepatocellular carcinoma [15–18]. However, its application in the context of CVC remains largely unexplored. This study aims to address this gap by employing the LASSO regression model to evaluate incident and prognostic risk factors in CVC patients. By leveraging this advanced statistical approach, we seek to identify a parsimonious set of variables most strongly associated with disease progression and overall survival in CVC. The implementation of LASSO-Cox regression in this context offers several advantages. First, it allows for effective variable selection in high-dimensional datasets, mitigating the risk of overfitting and enhancing model generalizability. Second, it can reveal complex interactions between predictors that may not be apparent in traditional regression analyses. Third, the LASSO method’s inherent feature selection capability aligns well with the goal of developing clinically applicable prognostic tools. This research has the potential to provide valuable insights into the clinical course of CVC, facilitating more accurate prognostication and informing the development of personalized treatment strategies. Moreover, by focusing on sex-specific patterns, this study may uncover important sex-based differences in CVC progression and outcomes. The results of this investigation may not only enhance our understanding of CVC but also demonstrate the broader applicability of the LASSO model in rare and understudied malignancies. This could establish a precedent for its use as a robust tool for prognostic modeling in clinical oncology, particularly in the context of sex-associated disparities in disease presentation and outcomes.

## Methods

### Search strategy and data collection

We acquired patient data from the publicly available Surveillance, Epidemiology, and End Results (SEER) database, which includes data from all 18 cancer registries available in the SEER 18 Regs Research Data (2000–2015) dataset accessible at www.seer.cancer.gov [19]. SEER*Stat version 8.3.6 software was employed to extract and analyze this data [20]. To comply with ethical and legal guidelines, efforts are underway to enhance access to the SEER Plus database. Within this context, our analysis focused on the publicly accessible SEER database, a comprehensive resource that covers roughly 28% of the North American population. From this extensive repository, we extracted pertinent data on patients diagnosed with CVC [21]. We subsequently identified the primary sites of CVC using the ICD-O-3 (International Classification of Diseases for Oncology, Third Edition) histological and behavioral codes ranging from ‘C00.0 to C63.2,’ including 8051/3 (Verrucous carcinoma, NOS). The main objective of this study is to identify factors associated with the prediction of CVC complications and survival outcomes using LASSO model in patients diagnosed between 2004 and 2015, as classified by the American Joint Commission on Cancer (AJCC) Sixth Edition staging system from 2004. Since cancer reporting is mandatory in all U.S. states, patient consent was not required. Upon signing a data usage agreement, cancer research data became publicly accessible. This study adheres to the STROCSS reporting guidelines.

### Data collection

**Fig. 1:**
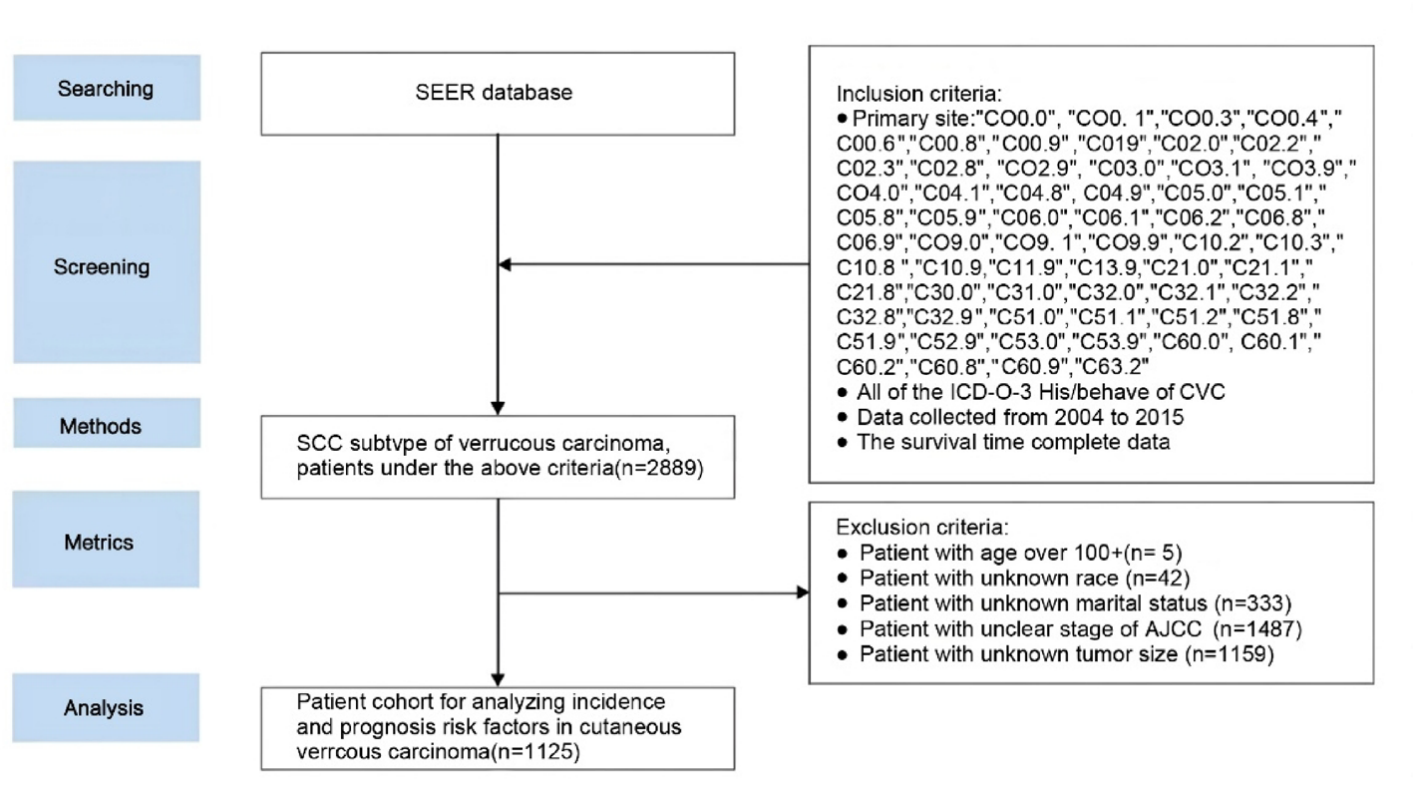
Literature screening and patient selection process of the study

The screening process involves a comprehensive range of demographic and clinical variables that serve as prognostic factors for CVC. These variables comprise age, sex, race, marital status, AJCC staging, tumor size (TS), income, as well as treatment modalities such as surgical interventions, radiation therapy, and chemotherapy. Utilizing the SEER database for a retrospective analysis, an initial cohort of 2,889 CVC patients from 2004 to 2015 was identified based on these criteria. After thorough evaluation, a subset of 1,125 patients met the final inclusion criteria, while 1,764 patients were excluded due to insufficient data on tumor size, race, marital status, AJCC stage, or age exceeding 100 years. Fig. 1 provides a graphical representation of the data selection process.

### LASSO regression

LASSO regression incorporates a penalty term that is applied to the sum of absolute values of the coefficients in the regression model. This penalty term effectively controls the number of parameters involved. In this context, the expression for the sum of squared error estimates for the Lasso model is given by,

$$\sum\limits_{{i = 1}}^{n} {\left( {y_{i} - \sum\limits_{j} {x_{{ij}} \beta _{j} } } \right)^{2} } + \lambda \sum\limits_{{j = 1}}^{p} {\left| {\beta _{j} } \right|} .$$

Here, N represents the number of observations, p denotes the predictor variables, y signifies the observed value for the ith observation, x stands for the value of the jth predictor, β0 is the intercept term, and βj represents the coefficient for the ith predictor. For the lasso regression analysis, we employed the ‘glmnet’ software package within the R programming language for both data processing and analysis [22]. These analyses were conducted using R version 4.3.0, in conjunction with the Storm Statistical Platform (www.medsta.cn/software) and SPSS (Chicago, Illinois, USA).

### Cox regression analyses of variables

To conduct a comprehensive analysis, we employed both univariate and multivariate Cox proportional hazard regression models to evaluate factors influencing survival rates in CVC patients. Variables demonstrating statistical significance in the univariate Cox regression were subsequently examined in the multivariate Cox regression model. Multivariate proportional hazard models were utilized to identify independent prognostic factors associated with CVC, along with their hazard ratios and 95% confidence intervals. A P-value < 0.05 was considered statistically significant. Statistical analyses were performed using SPSS software version 23.0 (Chicago, IL, USA), which included both Chi-square tests and Cox regression analysis to streamline the statistical evaluation process. For the multivariate Cox regression analysis of CVC outcomes, the independent prognostic factors assessed included age, sex, race, marital status, AJCC stage, combined summary stage, radiation, surgery, tumor size, regional lymph node involvement, and income.

### Kaplan-Meier (K-M) analysis

We conducted Kaplan-Meier (K-M) analysis, generating survival curves and performing a two-tailed log-rank test to compare variables demonstrating P values < 0.05 [23]. This approach effectively identifies and illustrates associations between continuous predictor variables and outcome measures.

### Restricted cubic spline analysis

Lastly, we employed the RCS as a predominant analytical approach, widely used to explore nonlinear relationships [24, 25]. Population characteristics were compared and classified using the Chi-square test, while continuous variables were presented as means (accompanied by standard deviations), and comparisons were made through t-tests. These methodologies formed the foundation for examining the results.

### Computational analysis and software integration

The R code used for LASSO regression and model evaluation has been included as supplementary material. Our analysis involved a multi-platform approach, integrating SEER*Stat version 8.3.6 for initial data extraction, R version 4.3.0 for advanced statistical modeling (particularly LASSO regression using the ‘glmnet’ package), the Storm Statistical Platform for data visualization, and SPSS version 23.0 for descriptive statistics and Cox regression analysis. Data transfer between platforms was facilitated using CSV files to ensure compatibility and maintain data integrity throughout the analysis pipeline. This integrated approach allowed us to leverage the strengths of each software platform while maintaining consistency in our analytical process. The LASSO-Cox model, implemented in R, was crucial for identifying key prognostic factors in CVC patients, offering a robust method for variable selection and shrinkage within the Cox proportional hazards model. This approach has enabled us to develop a parsimonious set of variables strongly associated with disease progression and overall survival in CVC, potentially informing more personalized treatment strategies and enhancing prognostic accuracy in clinical settings.

## Results

### Study cohort baseline characteristics

Between 2004 and 2015, this study included 1125 patients diagnosed with CVC, comprising 668 males (59.4%) and 457 females (40.6%) at initial diagnosis (Table 1). The study population was predominantly white, accounting for 949 cases (84.4%), with 556 males (83.2%) and 393 females (86.0%). Among the cohort, 584 patients (51.9%) were married, including 409 males (61.2%) and 175 females (38.3%). The distribution of American Joint Committee on Cancer (AJCC) stages showed a predominance in stage I, with 561 cases (49.9%), consisting of 374 males (56.0%) and 187 females (40.9%). Most cases were localized, representing 853 cases (78.5%), with 532 males (79.6%) and 321 females (70.2%). The most common tumor size was 11–20 mm, seen in 226 males (33.8%) and 177 females (38.7%).

**Table 1:** Baseline characteristics of newly diagnosed CVC patients by sex

Variable	*N* (%)	Male	Female	*P*-value
N	1125	668	457	
Age				
Continuation	66.48 (14.56)	62.63 (13.81)	72.10 (13.80)	< 0.001
Race				
White	949 (84.4)	556 (83.2)	393 (86.0)	0.017
Black	105 (9.3)	75 (11.2)	30 (6.6)	
Other	71 (6.3)	37 (5.5)	34 (7.4)	
Marital status				
Single	220 (19.6)	153 (22.9)	67 (14.7)	< 0.001
Married	584 (51.9)	409 (61.2)	175 (38.3)	
Divorced/widowed/separated	321 (28.5)	106 (15.9)	215 (47.0)	
AJCC stage				
I	561 (49.9)	374 (56.0)	187 (40.9)	< 0.001
II	340 (30.2)	174 (26.0)	166 (36.3)	
III	136 (12.1)	77 (11.5)	59 (12.9)	
IV	88 (7.8)	43 (6.4)	45 (9.8)	
Combined summary stage				
Local	853 (75.8)	532 (79.6)	321 (70.2)	0.001
Regional	214 (19.0)	110 (16.5)	104 (22.8)	
Distant	58 (5.2)	26 (3.9)	32 (7.0)	
Radiation				
No/unknown	892 (79.3)	519 (77.7)	373 (81.6)	0.128
Yes	233 (20.7)	149 (22.3)	84 (18.4)	
Chemotherapy				
No/unknown	1026 (91.2)	608 (91.0)	418 (91.5)	0.878
Yes	99 (8.8)	60 (9.0)	39 (8.5)	
Surgery				
No/unknown	118(10.5)	75(11.2)	43(9.4)	0.328
Yes	1007(89.5)	593(88.8)	414(90.6)	
Tumor size				
1–10 mm	137 (12.2)	81 (12.1)	56 (12.3)	0.016
11–20 mm	403 (35.8)	226 (33.8)	177 (38.7)	
20–40 mm	221 (19.6)	121 (18.1)	100 (21.9)	
> 40 mm	364 (32.4)	240 (35.9)	124 (27.1)	
Regional lymph node positive				
No	166 (14.8)	87 (13.0)	79 (17.3)	0.058
Yes	959 (85.2)	581 (87.0)	378 (82.7)	
Income				
<$35,000, $35, 000–44,999	113 (10.0)	72 (10.8)	41 (9.0)	0.764
$45,000-$59,999	271 (24.1)	161 (24.1)	110 (24.1)	
$60,000–74,999	473 (42.0)	280 (41.9)	193 (42.2)	
$75,000+	268 (23.8)	155 (23.2)	113 (24.7)	

### Analysis of incidence in patients with CVC

The median age at diagnosis was 68.25 ± 13.96 years, with a notable difference between males (64.17 ± 12.98 years) and females (72.85 ± 13.61 years) (Table 2). Marital status emerged as a significant factor, with married individuals exhibiting a 4.6-fold higher predicted risk of CVC complications compared to their unmarried counterparts, according to our LASSO logistic regression model. Disease staging analysis demonstrated a predominance of AJCC stage I cases, with a statistically significant sex difference (males 58.66% vs. females 41.34%, *P* < 0.05). Similarly, localized tumors were more prevalent in males (56.42% vs. 43.57% in females, *P* < 0.05). Radiation treatment was more frequently administered to male patients with CVC compared to females (*P* < 0.05), possibly indicating a sex-based difference in disease presentation or treatment approach. Surgical intervention rates were comparable between sexes, with 51.50% of males and 48.49% of females receiving surgical treatment. Interestingly, lymph node metastasis was more frequently observed in female patients (57.22%) (*P* < 0.05). The remaining variables showed no statistically significant differences between sex (Table 2). These findings provide valuable insights into the sex-specific patterns of CVC presentation and treatment, potentially informing tailored clinical approaches and future research directions in this field.

**Table 2:** Sex wise baseline characteristics and incidence rates of patients with CVC

Variable	Tumor patient *N*	Ratio (%)	Male	Female	*P*-value
Age					
Continuation	68.25 ± 13.96	-	64.17 ± 12.98	72.85 ± 13.61	< 0.01
Race					
White	1114 (86.49)	0.906	587 (52.69)	527 (47.307)	0.173
Black	92 (6.865)	0.892	42 (45.652)	50 (54.348)	
Other	89 (6.872)	0.8511	53 (59.551)	36 (40.449)	
Marital status					
Single	57 (11.899)	1.62	35 (61.403)	22 (38.596)	< 0.01
Married	210 (43.841)	2.0481	140 (66.666)	70 (33.333)	
Divorced/widowed/separated	212 (44.258)	0.3587	55 (25.943)	157 (74.0566)	
AJCC stage					
I	491 (48.613)	1.45	288 (58.6558)	203 (41.344)	0.0017
II	312 (30.891)	0.866	143 (45.833)	169 (54.166)	
III	125 (12.376)	1.04	63 (50.4)	62 (49.6)	
IV	82 (8.118)	0.842	37 (45.122)	45 (54.878)	
Combined summary stage					
Local	778 (76.953)	1.326	439 (56.426)	339 (43.573)	0.00037
Regional	173 (17.111)	0.73	72 (41.618)	101 (58.381)	
Distant	60 (5.934)	0.7315	25 (41.666)	35 (58.333)	
Radiation					
No/unknown	804 (79.525)	1.063	409 (50.871)	395 (49.129)	0.007
Yes	207 (20.474)	1.525	127 (61.353)	80 (38.647)	
Chemotherapy					
No/unknown	941 (93.076)	1.156	499 (53.0286)	442 (46.971)	0.9778
Yes	70 (6.9238)	1.482	37 (52.857)	33 (47.143)	
Surgery					
No/Unknown	116 (11.451)	1.945	76 (65.517)	40 (34.4827)	0.00443
Yes	897 (88.548)	1.0876	462 (51.505)	435 (48.494)	
Regional lymph node positive					
No	838 (82.888)	1.2583	462 (55.131)	376 (44.868)	0.00302
Yes	173 (17.111)	0.7654	74 (42.774)	99 (57.225)	
Income					
<$35,000, $35, 000–44,999	65 (6.493)	1.442	38 (58.461)	27 (41.538)	0.5791
$45,000-$59,999	220 (21.978)	1.298	123 (55.909)	97 (44.0901)	
$60,000–74,999	381 (38.619)	1.183	204 (53.543)	177 (48.955)	
$75,000+	335 (33.466)	1.067	171 (51.044)	164 (48.955)	
City	841 (83.184)	1.1045	436 (51.843)	405 (48.157)	0.0962
Rural	170 (16.815)	1.4404	100 (58.823)	70 (41.767)	

### Predictive variable selection for the Lasso based logistic regression model

**Fig. 2:**
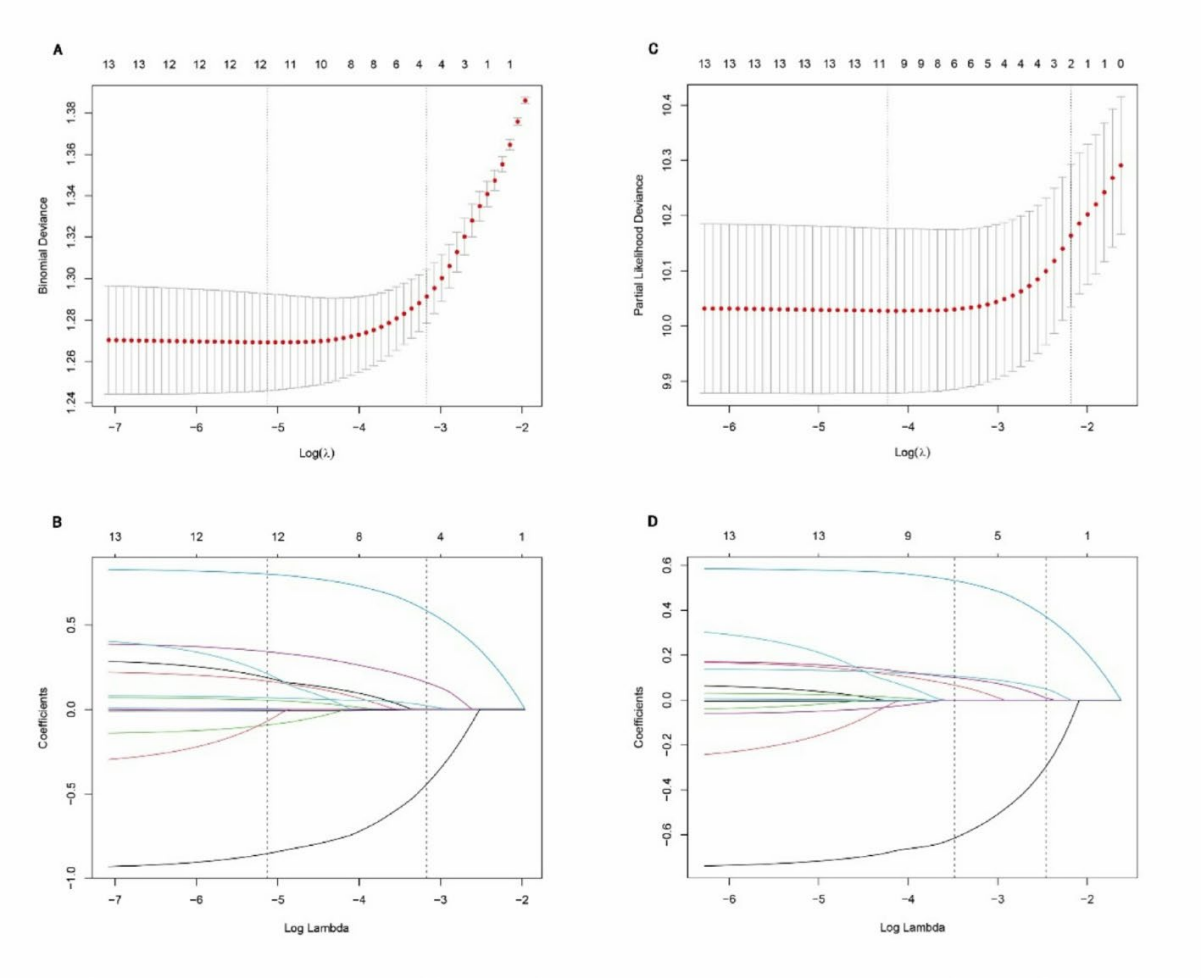
The variable selection of the lasso regression. **A** LASSO based logistic regression coefficient profiles of the 12 variables. A vertical line was drawn at the value chosen by cross-validation. As the value of λ decreased, the degree of model compression increased and the function of the model to select important variables increased. **B** The cross-validation results. The value in the middle of the two dotted lines is the range of the positive and negative standard deviations of log (λ). The dotted line on the left indicated the value of the harmonic parameter log (λ) when the error of the model is minimized. Eleven variables were selected when log (λ) = 0.0059. **C** The partial likelihood deviance of various combinations of variables calculated by the LASSO-cox regression analysis. **D** Six risk factors selected using LASSO Cox regression analysis. The two dotted vertical lines were drawn at the optimal scores by minimum criteria and 1-s.e. criteria (At minimum criteria including age, sex, marital status, AJCC stage, combined summary stage, surgery)

We developed a risk prediction model for CVC complications utilizing LASSO logistic regression to enhance predictive accuracy and mitigate overfitting. In this model, we defined the dependent variable as a binary outcome and executed the LASSO regression using the glmnet function with the family = binomial parameter. The variable selection process associated with CVC risk is detailed in Supplementary Table 1. To optimize the model, we determined the optimal lambda value (λ = 0.0129) through cross-validation, which allowed us to fine-tune the model and evaluate the potential contributions of each predictor (Fig. 2A). This process resulted in the shrinkage of coefficients for 12 variables to zero, yielding a parsimonious final model comprising 11 significant variables: Age, Sex, Race, Marital Status, American Joint Committee on Cancer (AJCC) Stage, Combined Summary Stage, Radiation Treatment, Surgery, Tumor Size, Chemotherapy, and Regional Lymph Node Involvement (Fig. 2B and Supplementary Table 2). The LASSO regression’s inherent feature selection capability enabled the identification of these key predictors, each demonstrating a significant association with the binary outcome. This approach not only enhances the model’s interpretability but also potentially improves its generalizability by reducing the risk of overfitting. The selected variables represent a comprehensive set of demographic, clinical, and treatment-related factors that collectively contribute to the prediction of CVC complications.

### Predictive variable selection for the Lasso based Cox regression model

The LASSO-Cox model was employed to analyze 12 variable assignments (Supplementary Table 1) in CVC patients, enhancing the efficiency of predictor selection. This advanced statistical approach effectively reduced the number of independent variables to six potential predictors. The risk factors identified by the LASSO-Cox regression model are presented in Supplementary Table 3. Fig. 2C, D illustrate the results of the selected independent variables derived from the LASSO-based Cox regression, providing a visual representation of their relative importance. Following comprehensive analysis, the LASSO-Cox model identified six key risk factors, age, sex, marital status, AJCC stage, combined summary stage, and surgery. These factors emerged as the most significant predictors of outcomes in CVC patients, offering a refined and statistically robust set of variables for prognostic assessment in clinical practice.

### Correlations of independent clinical risk features for CVC using lasso-logistic regression0.327

**Fig. 3:**
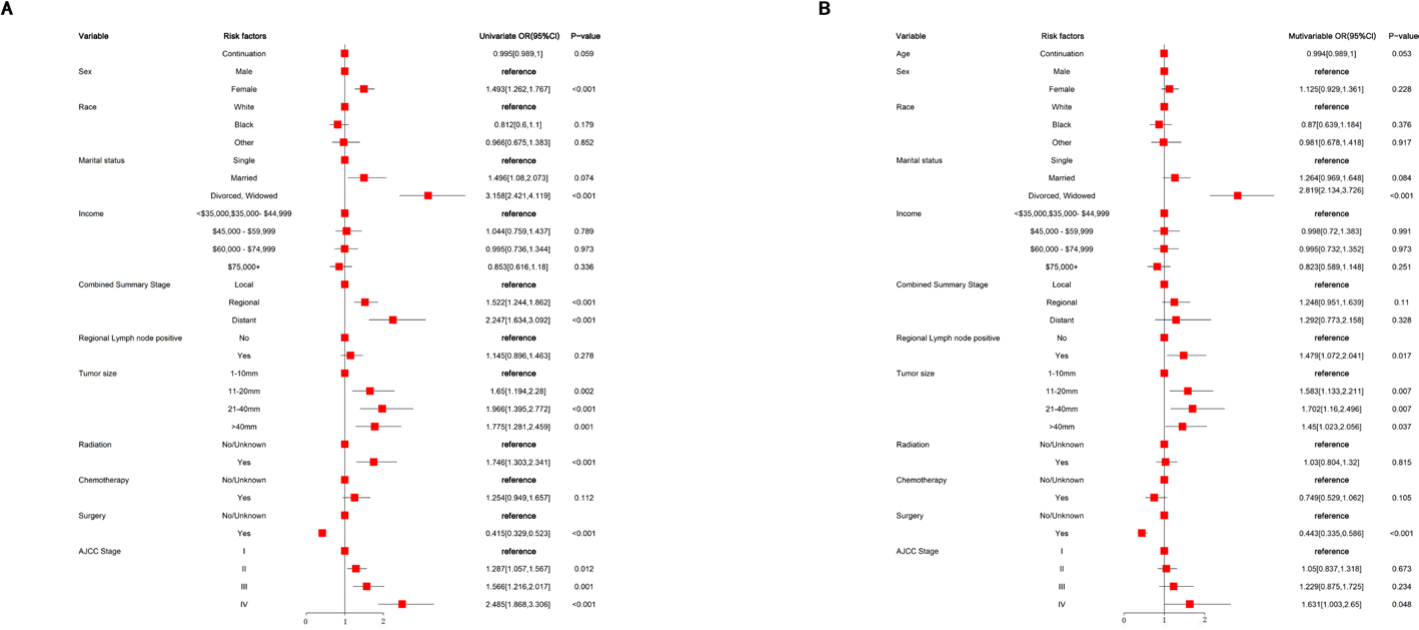
Forest plot of the uni variant and multi variant lasso-logistic regression model evaluating incident of CVC

A comprehensive univariate logistic regression analysis was conducted to identify potential factors associated with the prediction of CVC complications using the LASSO model, as detailed in Table 3; Fig. 3. This analysis revealed several statistically significant variables associated with the LASSO prediction of CVC complications, including age (OR = 0.99, 95% CI 0.982–0.998, *P* = 0.017), sex (*P* < 0.001), marital status ((all *P* < 0.05)), AJCC stage (all *P* < 0.05), region (all *P* < 0.05), radiation therapy (*P* < 0.05), surgical treatment (*P* < 0.001), and tumor size (all *P* < 0.05) (Table 3). These variables were subsequently incorporated into a multivariate logistic regression model for further analysis. The multivariate logistic regression analysis yielded significant insights into factors impacting the LASSO-predicted risk of CVC complications. Marital status emerged as a significant predictor, with married individuals demonstrating a higher risk compared to their single counterparts (OR = 1.536, 95% CI 1.09–2.614, *P* = 0.014). Surgical intervention was associated with a protective effect (OR = 0.382, 95% CI 0.232–0.63, *P* < 0.001). Tumor size exhibited a dose-response relationship with CVC risk, tumors measuring 11–20 mm (OR = 1.986, 95% CI 1.276–3.092, *P* = 0.002), 20–40 mm (OR = 2.174, 95% CI 1.274–3.708, *P* = 0.004), and exceeding 40 mm (OR = 1.715, 95% CI 1.074–2.737, *P* = 0.024) all conferred significantly higher risks compared to tumors 1–10 mm in diameter (Table 3). These findings provide valuable insights into the complex interplay of factors influencing the LASSO-predicted risk of CVC complications and their progression.

**Table 3:** Univariate and multivariate lasso-logistic regression model for the incident of the CVC

Variable	OR(univariable)		OR(multivariable)	
	OR(95%CI)	P-value	OR(95%CI)	P-value
Age				
Continuation	0.99 (0.982,0.998)	0.017	0.988 (0.979,0.997)	0.008
Sex				
Male	Reference		Reference	
Female	1.66 (1.31,2.12)	< 0.001	1.15 (0.87,1.52)	0.327
Race				
White	Reference		Reference	
Black	0.795 (0.529,1.192)	0.267	0.801 (0.516,1.242)	0.321
Other	0.836 (0.515,1.357)	0.469	0.785 (0.456,1.353)	0.384
Marital status				
Single	Reference		Reference	
Married	1.496 (1.08,2.073)	0.015	1.536 (1.09,2.164)	0.014
Divorced/widowed/separated	5.195 (3.582,7.535)	< 0.001	4.976 (3.334,7.425)	< 0.001
AJCC stage				
I	Reference		Reference	
II	1.347 (1.028,1.766)	0.031	0.995 (0.712,1.391)	0.976
III	1.925 (1.316,2.815)	0.001	1.24 (0.735,2.091)	0.42
IV	2.741 (1.705,4.407)	< 0.001	0.987 (0.449,2.171	0.974
Combined summary stage				
Local	Reference		Reference	
Regional	1.782 (1.315,2.415)	< 0.001	1.462 (0.958,2.23)	0.078
Distant	3.267 (1.809,5.903)	< 0.001	2.72 (1.112,6.654)	0.028
Radiation				
No/unknown	Reference		Reference	
Yes	1.746 (1.303,2.341)	< 0.001	1.386 (0.931,2.065)	0.108
Chemotherapy				
No/unknown	Reference		Reference	
Yes	1.351 (0.892,2.046)	0.155	0.734 (0.421,1.282)	0.277
Surgery				
No/unknown	Reference		Reference	
Yes	0.33 (0.217,0.503)	< 0.001	0.382 (0.232,0.63)	< 0.001
Tumor size				
1–10 mm	Reference		Reference	
11–20 mm	2.014 (1.341,3.025)	0.001	1.986 (1.276,3.092)	0.002
20–40 mm	2.342 (1.503,3.651)	< 0.001	2.174 (1.274,3.708)	0.004
> 40 mm	2.113 (1.4,3.189)	< 0.001	1.715 (1.074,2.737)	0.024
Regional lymph node positive				
No	Reference		Reference	
Yes	1.215 (0.872,1.692)	0.249	1.34 (0.922,1.949)	0.125
Income				
<$35,000, $35, 000–44,999	Reference		Reference	
$45,000-$59,999	1.235 (0.796,1.918)	0.347	1.168 (0.724,1.886)	0.524
$60,000–74,999	1.149 (0.761,1.733)	0.51	1.195 (0.761,1.876)	0.439
$75,000+	0.995 (0.64,1.547)	0.983	1.046 (0.644,1.698)	0.856

### Correlations of independent clinical prognostic factors for CVC by lasso-cox regression

**Fig. 4:**
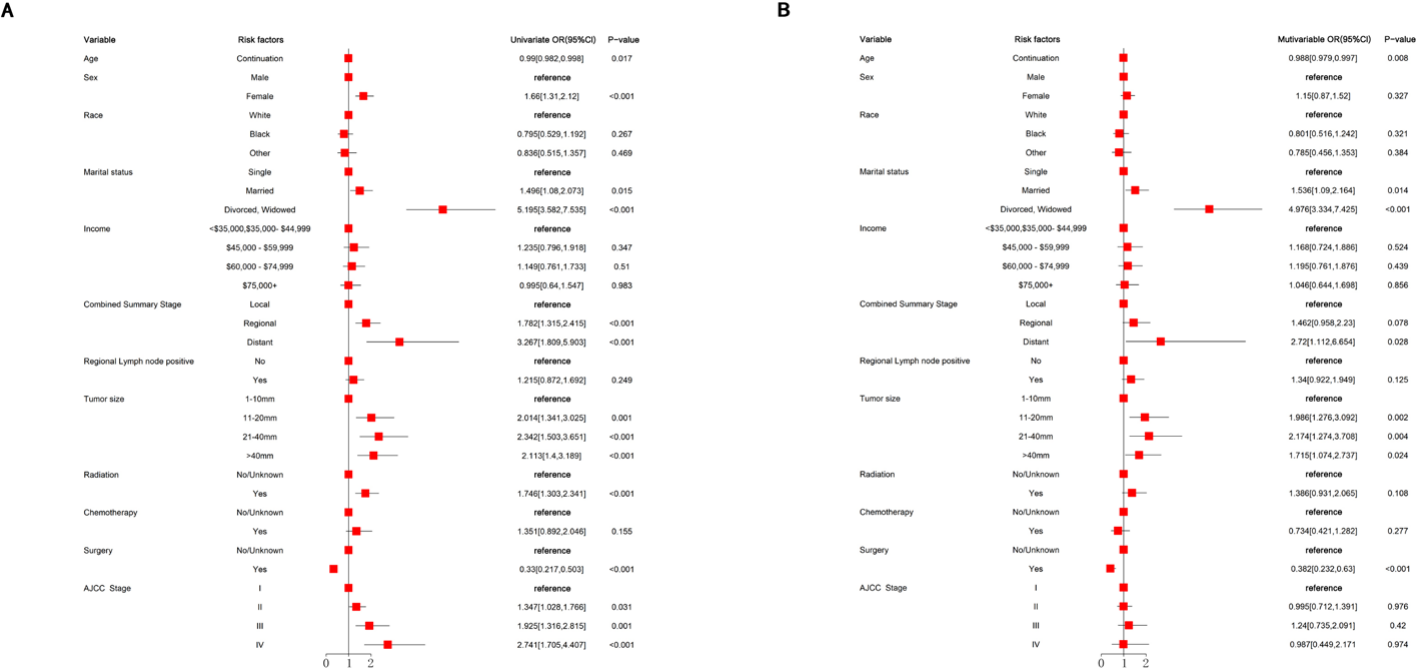
Forest plot of the lasso-cox regression model evaluating mortality of cutaneous verrucous carcinoma

The univariate Cox regression model revealed significant differences in OS among patients with CVC, as detailed in Table 4 and illustrated in Fig. 4. This analysis identified several independent prognostic factors affecting OS, including sex, AJCC stage, combined summary stage, radiation treatment, surgical intervention, and tumor size. Further refinement through multivariate Cox regression analysis identified specific factors independently associated with OS. Notably, surgical intervention was a significant protective factor, with patients who underwent surgery demonstrating a substantially reduced hazard ratio compared to those who did not receive surgical treatment (HR = 0.443, 95% CI 0.356–0.586, *P* < 0.001). Additionally, tumor size showed a dose-response relationship with OS; tumors measuring 11–20 mm in diameter had an increased risk compared to those measuring 1–10 mm (OR = 1.583, 95% CI 1.133–2.211, *P* = 0.007), while tumors sized 20–40 mm also exhibited a higher risk (OR = 1.702, 95% CI 1.160–2.496, *P* = 0.007). Tumors greater than 40 mm in size were associated with an increased risk as well (OR = 1.450, 95% CI 1.023–2.056, *P* = 0.037) (Table 4). These findings underscore the importance of surgical intervention and tumor size as critical factors influencing overall survival in CVC patients.

**Table 4:** Univariate and multivariate Lasso-Cox regression models for the incidence of the CVC

Variable	HR (univariable)		HR (multivariable)	
	HR (95%CI)	P-value	HR (95%CI)	P-value
Age	0.995 (0.989,1)	0.059	0.994 (0.989,1)	0.053
Continuation				
Sex				
Male	Reference		Reference	
Female	1.493 (1.262,1.767)	0.000	1.125 (0.929,1.361)	0.228
Race				
White	Reference		Reference	
Black	0.812 (0.6,1.1)	0.179	0.87 (0.639,1.184)	0.376
Other	0.966 (0.675,1.383)	0.852	0.981 (0.678,1.418)	0.917
Marital status				
Single	Reference		Reference	
Married	1.496 (1.08,2.073)	0.074	1.264 (0.969,1.648)	0.084
Divorced/widowed/separated	3.158 (2.421,4.119)	0	2.819 (2.134,3.726)	< 0.001
AJCC stage				
I	Reference		Reference	
II	1.287 (1.057,1.567)	0.012	1.05 (0.837,1.318)	0.673
III	1.566 (1.216,2.017)	0.001	1.229 (0.875,1.725)	0.234
IV	2.485 (1.868,3.306)	< 0.001	1.631 (1.003,2.65)	0.048
Combined summary stage				
Local	Reference		Reference	
Regional	1.522 (1.244,1.862)	0	1.248 (0.951,1.639)	0.11
Distant	2.247 (1.634,3.092)	0	1.292 (0.773,2.158)	0.328
Radiation				
No/unknown	Reference		Reference	
Yes	1.746 (1.303,2.341)	< 0.001	1.03 (0.804,1.32)	0.815
Chemotherapy				
No/unknown	Reference		Reference	
Yes	1.254 (0.949,1.657)	0.112	0.749 (0.529,1.062)	0.105
Surgery				
No/unknown	Reference		Reference	
Yes	0.415 (0.329,0.523)	0	0.443 (0.335,0.586)	< 0.001
Tumor size				
1–10 mm	Reference		Reference	
11–20 mm	1.65 (1.194,2.28)	0.002	1.583 (1.133,2.211)	0.007
20–40 mm	1.966 (1.395,2.772)	0	1.702 (1.16,2.496)	0.007
> 40 mm	1.775 (1.281,2.459)	0.001	1.45 (1.023,2.056)	0.037
Regional lymph node positive				
No	Reference		Reference	
Yes	1.145 (0.896,1.463)	0.278	1.479 (1.072,2.041)	0.017
Income				
<$35,000, $35, 000–44,999	Reference		Reference	
$45,000-$59,999	1.044 (0.759,1.437)	0.789	0.998 (0.72,1.383)	0.991
$60,000–74,999	0.995 (0.736,1.344)	0.973	0.995 (0.732,1.352)	0.973
$75,000+	0.853 (0.616,1.18)	0.336	0.823 (0.589,1.148)	0.251

### The prognostic of CVC patients based on Kaplan–Meier method

**Fig. 5:**
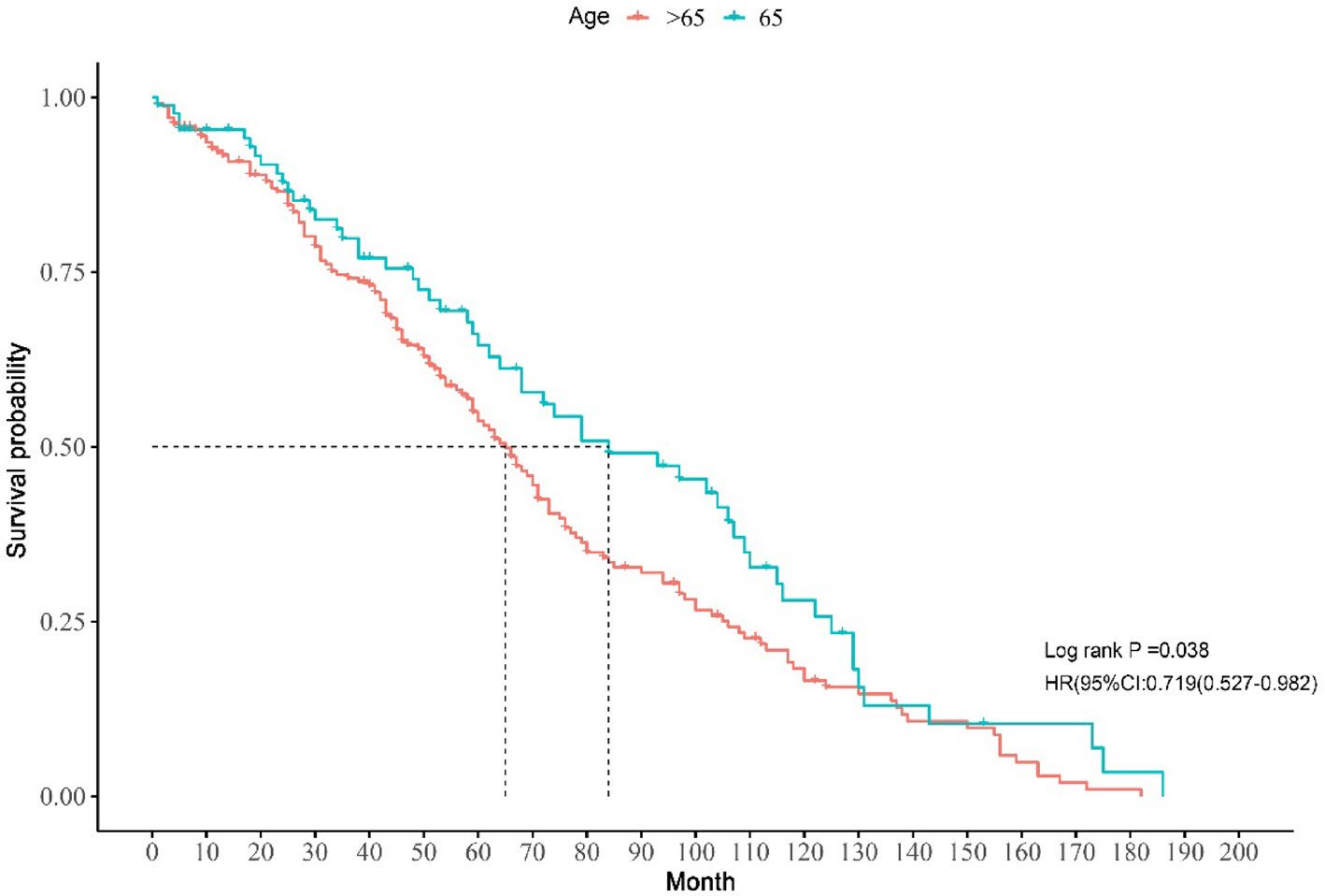
Kaplan-Meier chart illustration of various independent variable in the survival curves for cutaneous verrucous carcinoma

We conducted a comprehensive analysis utilizing Kaplan-Meier (KM) survival curves to evaluate the impact of various independent variables on the overall survival of patients with CVC. This examination revealed statistically significant correlations with specific variables, including sex, radiation exposure, and surgical interventions. Inspection of the Kaplan-Meier curves demonstrated that the female cohort exhibited a less favorable prognosis compared to their male counterparts. Quantitatively, the median survival for males was 150 months, significantly higher than the 87 months observed for females. This stark contrast in survival outcomes underscores the importance of sex as a prognostic factor in CVC. Fig. 5 provides a detailed visual representation of these findings, illustrating the overall survival trends captured by the KM curves. These curves are stratified by sex and further segmented into different patient groups based on other relevant clinical factors. This graphical representation allows for a significant interpretation of survival patterns across various sub populations within the CVC patient cohort. The observed differences in survival outcomes between male and female patients suggest potential underlying biological or treatment-related factors that may influence disease progression and response to therapy in CVC. These findings highlight the need for further investigation into sex-specific risk factors and potentially tailored treatment approaches for CVC patients.

### Sex wised disparities median survival period in CVC patients

**Fig. 6:**
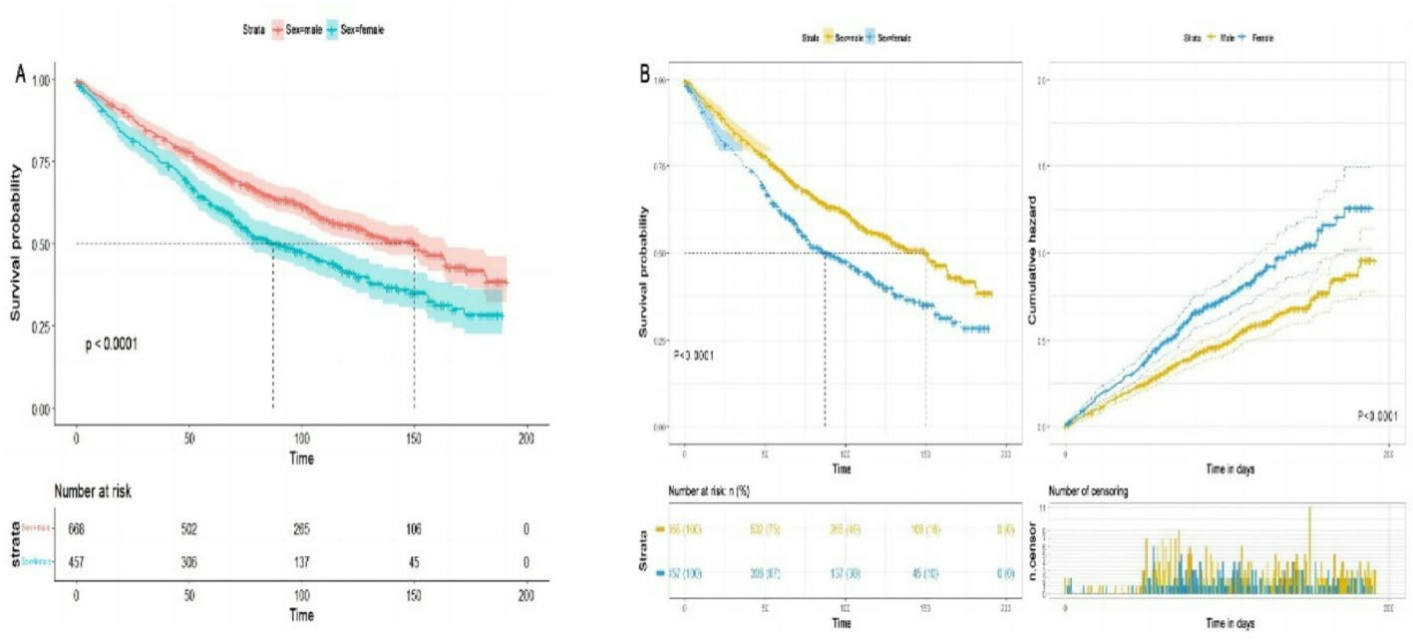
Kaplan-Meier chart illustration of sex disparity in the survival curves for cutaneous verrucous carcinoma

**Fig. 7:**
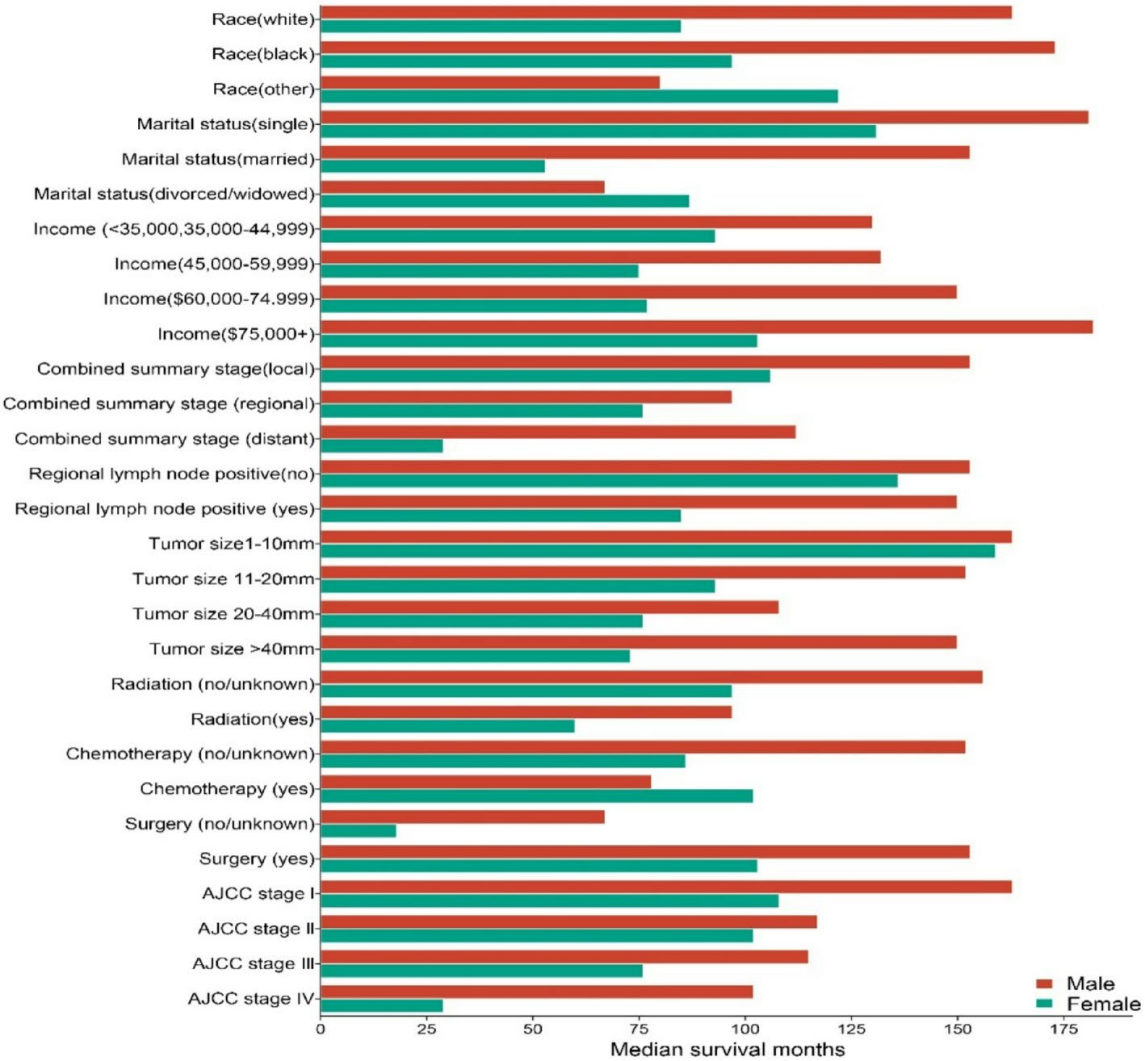
Plot Bar chart illustration sex disparity in median survival period of patients with cutaneous verrucous carcinoma

The Kaplan-Meier curves in this study revealed that female CVC patients had shorter OS compared to their male counterparts. The log-rank test confirmed a statistically significant difference in survival rates between male and female CVC patients (*P* < 0.05). sex-based disparities in median survival times for CVC patients are detailed in Table 5, Figs. 6 and 7. Specifically, white male patients had a median survival of 139 months, whereas female patients had a median survival of 85 months (*P* = 0.020). Male patients demonstrated longer median survival times compared to females among CVC patients. Statistically significant independent factors affecting survival outcomes included race, marital status, AJCC Stage IV, combined summary stage, radiation, surgery, tumor size, regional lymph node involvement, and income. Notably, among those receiving chemotherapy, female patients exhibited longer median survival times than their male counterparts.

**Table 5:** Sex disparity in median survival period of patients with cutaneous verrucous carcinoma

Variable	Sex		*P*-value
	Male (95%CI)	Female (95%CI)	
Race			
White	139 (127,163)	85 (73,107)	0.020
Black	173 (93,252)	97 (54,103)	0.716
Other	80 (56,111))	122 (97, 146)	0.678
Marital status			
Single	181 (164,205)	131 (105,156)	0.873
Married	153 (132,173)	53 (44,61)	0.977
Divorced/widowed/separated	67 (49,109)	87 (70,103)	0.498
AJCC stage			
I	163 (139, 186)	108 (90,136)	0.118
II	117 (80, 153)	102 (70,172)	0.705
III	115 (93, 136)	76 (50,100)	0.181
IV	102 (41,162)	29 (14,57)	0.359
Combined summary stage			
Local	153 (137,182)	106 (86,130)	0.073
Regional	97 (64,156)	76 (54,116)	0.477
Distant	112 (67,156)	29 (16,57)	0.396
Radiation			
No/unknown	156 (138,182)	97 (77,118)	0.013
Yes	97 (73,150)	60 (39,115)	0.498
Chemotherapy			
No/unknown	152 (129, 164)	86 (74,108)	0.015
Yes	78 (55, 166))	102 (46,157)	0.812
Surgery			
No/unknown	67 (53,137)	18 (9,35)	0.185
Yes	153 (132,181)	103 (85,124)	0.0345
Tumor size			
1–10 mm	163 (140,185)	159 (116,132)	0.674
11–20 mm	152 (120,183)	93 (73,118)	0.149
20–40 mm	108 (66,149)	76 (55,148)	0.515
> 40 mm	150 (117, 182)	73 (51,115)	0.063
Regional lymph node positive			
No	153 (112–193)	136 (90–181)	0.590
Yes	150 (127,165)	85 (73,105)	0.018
Income			
<$35,000, $35, 000–44,999	130 (102,157)	93 (53,154)	0.459
$45,000-$59,999	132 (96,167)	75 (60,167)	0.552
$60,000–74,999	150 (113,165)	77 (69,111)	0.193
$75,000+	182 (131,168)	103 (79,136)	0.037

### Age relationship between incidents CVC

**Fig. 8:**
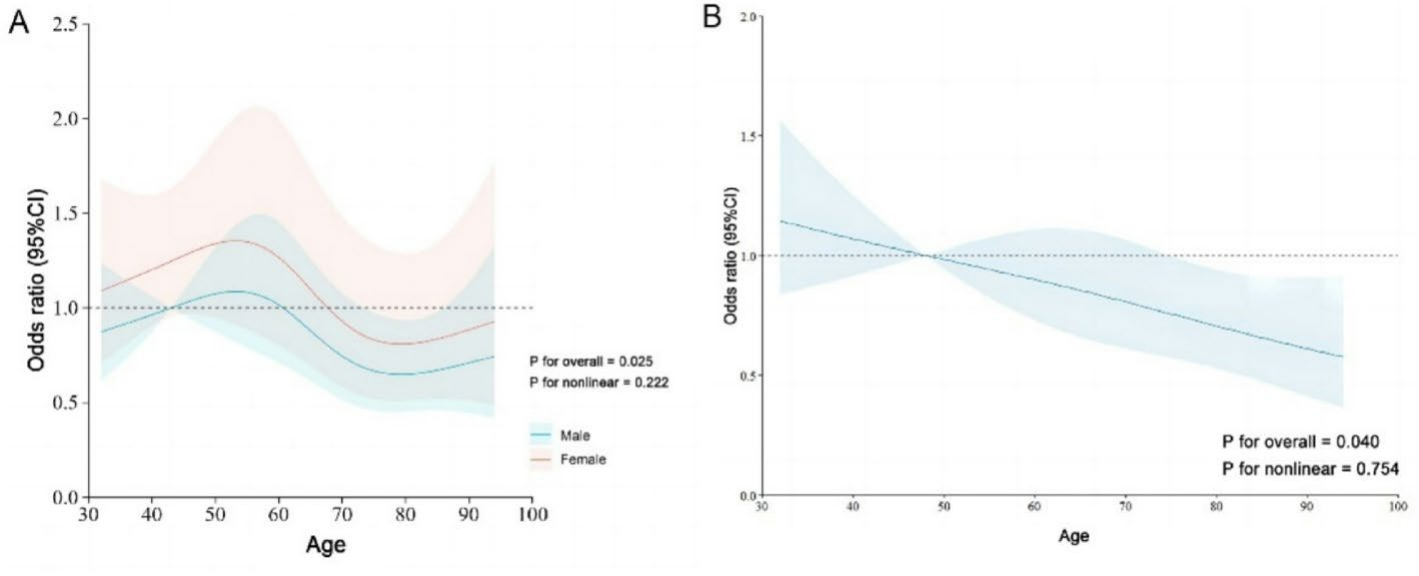
Correlated between age and incident of CVC patient using a restricted cubic spline regression model. **A** Correlated between age and incident of CVC patient using a restricted cubic spline regression model in sex disparities. **B** Age association with incident of CVC patient using a restricted cubic spline regression model in multivariable analysis

We employed a RCS model with four knots to explore the relationship between age and the incidence of CVC using age stratification. The analysis revealed a U-shaped correlation between age and all-cause CVC incidence, indicating a significant nonlinear relationship. Specifically, the risk of CVC increased sharply up to approximately age 67, after which it gradually declined. However, when a multivariable analysis was conducted using the RCS model, no nonlinear relationship between age and CVC incidence was observed (*P* = 0.754), suggesting no significant association between patient age and the multivariate incidence of CVC. Fig. 8 illustrates the absence of a nonlinear association between sex disparities and CVC incidence across various age groups (*P* = 0.222). The RCS model demonstrated an increased incidence of CVC between the ages of 40 and 60, followed by a decrease from ages 60 to 80, with an upward trend observed in individuals over 80 years old. These findings highlight the complex dynamics of age-related risk factors in CVC incidence and underscore the importance of considering age as a potential variable in epidemiological studies of CVC.

## Discussion

The CVC is a rare subtype of squamous cell carcinoma characterized by its low-grade, well-differentiated, and slow-growing nature. Despite its capacity for aggressive local tissue invasion, metastasis is exceptionally uncommon, contributing to a generally favorable prognosis with a 5-year survival rate exceeding 75% [26]. The etiology of CVC is multifactorial, with risk factors including chronic exposure to carcinogens such as ultraviolet radiation and human papillomavirus (HPV), as well as immunosuppression [27]. Histopathological factors, including invasion depth, perineural involvement, and tumor size, have been associated with poorer outcomes [28]. Delayed diagnosis and incomplete excision can potentially increase the risk of recurrence and metastasis. Further research is imperative to elucidate the complex interplay of factors influencing CVC pathogenesis and progression. This study aims to develop a risk prediction model for CVC complications based on a comprehensive set of clinical indicators, including age, sex, race, marital status, AJCC stage, and tumor size, to assist healthcare professionals in formulating tailored treatment strategies.

Univariate logistic regression analysis revealed several significant independent risk factors for CVC. Key variables identified include age (OR = 0.99, 95% CI 0.982–0.998, *p* = 0.017), sex, marital status, AJCC stage, geographic region, radiation therapy, surgical intervention, and tumor size (all *p* < 0.05) (Table 3). These factors warrant further investigation through multivariate logistic regression analysis. Notably, married individuals exhibited higher odds of CVC compared to their single counterparts (OR = 1.536, 95% CI 1.09–2.614, *p* = 0.014) [29, 30], suggesting a potential association between marital status and CVC risk that merits further exploration. Surgical treatment emerged as a significant protective factor against CVC (OR = 0.382, 95% CI 0.232–0.63, *P* < 0.001) [31, 32], underscoring its crucial role in disease management (Table 3). Larger tumor sizes (11–20 mm and > 40 mm) were associated with increased odds of CVC compared to smaller tumors (1–10 mm) [33], emphasizing the importance of early detection and prompt intervention. These findings, derived from the LASSO-logistic regression model, highlight the critical importance of factors such as marital status, surgical intervention, and tumor size in the comprehensive management of CVC patients. Overall, these results suggested that a multifaceted approach, considering both clinical and social determinants, is essential for optimizing CVC risk management strategies.

The application of the LASSO-based Cox regression model for variable selection in CVC patients has significantly enhanced predictive efficiency. This approach is renowned for its ability to handle high-dimensional data, select relevant variables, and mitigate overfitting [34]. Our findings align with previous studies that have successfully employed the LASSO technique to identify key prognostic factors in similar clinical contexts [35–37]. The reduction of 12 initial variables to 6 key predictors through LASSO-based Cox regression is consistent with research highlighting the model’s strength in feature selection [38]. This streamlining process not only enhances the model’s interpretability but also augments its clinical utility, facilitating more informed decision-making [39]. The capability of LASSO-Cox model to report multicollinearity among variables provides an advantage over traditional univariate analysis, particularly valuable in clinical settings where multiple factors may interact complexly. By incorporating a wide range of indicators, the model provides a comprehensive evaluation of individual patient situations, allowing for more accurate prediction of relapse risk and potentially leading to improved patient outcomes through tailored follow-up strategies and treatment protocols.

The variables identified by the LASSO-Cox regression model align with established risk factors for CVC, including age, sex, marital status, AJCC stage, combined summary stage, and surgery. These findings are consistent with existing literature on prognostic factors in various cancers [40, 41]. Incorporating these key risk factors into the Lasso-Cox model enhances its predictive accuracy and highlights its potential clinical utility. Our study, by combining established risk factors with advanced modeling techniques, offers a strong framework for better prognostic assessments in CVC patients. Kaplan-Meier analysis identified significant prognostic factors for CVC, particularly sex, radiation exposure, and surgery. Males had a significantly better prognosis (median survival of 150 months) than females (87 months), suggesting potential biological or clinical influences that need further study. This sex-based variance in survival could be recognized to various factors, such as hormonal influences, differences in treatment responses, or variations in healthcare seeking behaviors. For instance, estrogen prothrombotic effects might predispose women to CVC related thrombosis, potentially contributing to poorer outcomes [42]. The effect of radiation exposure and surgery on prognosis highlights the importance of treatment modalities in CVC management. Surgical interventions protective effect probably relates to reduced tumor burden and decreased dependence on long-term CVC use. The influence of radiation therapy might be complex, possibly affecting vascular health and following CVC-related complications. Understanding these factors are important for optimizing treatment strategies and developing personalized risk assessment tools [43]. The survival trends illustrated in Fig. 5 underlines the importance of considering sex and other identified risk factors in treatment planning and follow up protocols for CVC patients. Further research should focus on elucidating the underlying mechanisms of these prognostic factors to inform targeted interventions and improve outcomes in CVC management.

Our study reveals a significant sex disparity in median survival, with female patients exhibiting notably shorter OS than their male counterparts, as confirmed by Kaplan-Meier curves and log-rank tests (*P* < 0.05). White male patients demonstrated a median survival of 139 months, compared to 85 months for female patients (*P* = 0.020), highlighting the critical role of sex in CVC management. Females are known to experience greater chemotherapy related toxicities, which could contribute to poor outcomes. Additionally, sex specific differences in immune responses and treatment tolerability may play a role, as females often exhibit higher susceptibility to adverse events from chemotherapy and immunotherapy. Additional factors, including race, marital status, AJCC Stage IV, combined summary stage, radiation therapy, surgical intervention, tumor size, lymph node involvement, and income, also significantly influenced survival outcomes (*P* < 0.05). Interestingly, in the subgroup of patients receiving chemotherapy, females demonstrated longer median survival times, indicating a potential sex-specific response to treatment that warrants further investigation. These findings underscore the importance of sex-inclusive approaches in prognosis and treatment planning, with the identified factors providing valuable insights for personalized care strategies.

Our analysis of the relationship between age and CVC incidence yielded several key insights. Utilizing a RCS model with four knots, we identified a U-shaped pattern, demonstrating a sharp increase in CVC risk up to age 67, followed by a gradual decline. However, when adjusting for multiple variables, the nonlinear association between age and CVC incidence disappeared (*P* = 0.754), suggesting that age alone may not be a significant factor in CVC risk within a multivariate framework. Additionally, we found no significant nonlinear association between sex disparities and CVC incidence across age groups (*P* = 0.222). The RCS model revealed a higher incidence of CVC between ages 40 and 60, a decline from 60 to 80, and a subsequent increase in individuals over 80 years of age. These findings highlight the need for age-specific prevention strategies and increased vigilance in CVC cancer screening across all age groups, particularly in the elderly population.

This study aimed to develop a comprehensive framework for the early identification of high-risk individuals for CVC, with a focus on personalized treatment strategies and guiding future research directions. The LASSO-logistic regression model identified key factors such as marital status, surgical intervention, and tumor size as crucial elements for evaluating and treating CVC patients. By integrating established risk factors with advanced modeling techniques, we provide a solid foundation for improving prognostic assessments in CVC. sex-inclusive strategies are essential for accurate prognosis and effective treatment, with our findings offering valuable insights for personalized care approaches. The RCS model highlights a higher CVC incidence between ages 40–60, a decline from 60 to 80, and an increase in individuals over 80 years of age.

However, this study has several limitations. The SEER database lacks detailed tumor information, potentially affecting the accuracy of prognostic assessments [44]. Not all cancer cases are screened, which may lead to underestimations of unscreened CVC metastasis. We were unable to assess initial CVC diagnoses or recurrences. Relying on a single dataset introduces potential bias, and the absence of prospective validation is a concern. To address these limitations, we plan to create a new model with more diverse samples and establish a prospective cohort for validation. Future research will explore other machine learning techniques, such as XGBoost and Support Vector Machines (SVM), to further refine our predictive models and enhance our understanding of CVC pathogenesis and progression.

## Electronic supplementary material

Below is the link to the electronic supplementary material.

Supplementary Material 1

## Data Availability

Publicly available datasets were analyzed in this study. This data can be found at: https://seer.cancer.gov/.

## Abbreviations

CVC: cutaneous verrucous carcinoma
AJCC: American Joint Committee on Cancer
SEER: Surveillance, Epidemiology, and End Results
cSCC: cutaneous squamous cell carcinoma
RCS: restricted cubic spline
K-M: Kaplan-Meier
