# Supplementary material for: Assessing the prognosis mortality in patients with cutaneous verrucous carcinoma using Lasso-cox regression model: a retrospective study
Source: Discov Oncol. 2025 Jun 13;16:1091. doi: 10.1007/s12672-025-02893-6 (PMC12165914; doi:10.1007/s12672-025-02893-6)
Supplement: Supplementary file 1 — Supplementary Material 1 [file 12672_2025_2893_MOESM1_ESM.docx]

**Supplementary data**

**Sup table 1:** Variable assignments.

| variable | Risk factors | Assignment |
| --- | --- | --- |
| X1 | Age | continuous variable |
| X2 | Sex | male = 1,  female= 2 |
| X3 | Race | White=1,  Black=2,  Other=3 |
| X4 | Marital status | Single=1,  Married=2,  Divorced, Widowed=3 |
| X5 | AJCC Stage | I=1,  II=2,  III=3,  IV=4 |
| X6 | Combined Summary Stage | Local=1,  Regional=2,  Distant=3 |
| X7 | Radiation | Yes=1,  No/Unknown=2 |
| X8 | Chemotherapy | Yes=1,  No/Unknown=2 |
| X9 | Surgery | Yes=1,  No/Unknown=2 |
| X10 | Tumor size | 1-10mm=1,  11-20mm=2,  20-40mm=3,  >40mm=4 |
| X11 | Regional Lymph node positive | Yes=1,  No=2 |
| X12 | Income | <$35,000, $35, 000-44,999=1,  $45,000-$59,999=2,  $60,000-74,999=3,  $75,000+=4 |

**Sup table 2:** Risk factors selected by lasso-logistic regression model

| variable | Risk factors | coefficient |
| --- | --- | --- |
| X_1_ | Age | -0.009039876 |
| X_2_ | Sex | 0.167176854 |
| X_3_ | Race | -0.092240089 |
| X_4_ | Marital status | 0.801130238 |
| X_5_ | AJCC Stage | 0.068755130 |
| X_6_ | Combined Summary Stage | 0.341809896 |
| X_7_ | Radiation | 0.187265904 |
| X8 | Chemotherapy | -0.068071659 |
| X9 | Surgery | -0.853311663 |
| X10 | Tumor size | 0.054364964 |
| X11 | Regional Lymph node positive | 0.210366714 |

**Sup table 3:** Risk factors selected by lasso-cox model

| variable | Risk factors | coefficient |
| --- | --- | --- |
| X1 | Age | -0.0009050134 |
| X2 | Sex | 0.0663886342 |
| X_4_ | Marital status | 0.5326224337 |
| X_5_ | AJCC Stage | 0.1070693575 |
| X_6_ | Combined Summary Stage | 0.1001525620 |
| X9 | Surgery | -0.6150975754 |

**R code:**

Baseline Tables (Table 1 and 2) were analyzed and exported using the Storm statistical platform.

Figure 2, The variable selection of the lasso regression plot, was created using R code:

# Set working directory

setwd("C:/Users/Dell/Desktop")

# Install and load required packages

install.packages("remotes")

remotes::install_github("mlr-org/mlr3proba")

library(mlr3)

library(mlr3proba)

library(dplyr)

library(mlr3verse)

library(ggplot2)

library(magrittr)

library(survival)

# Read data

mydata <- read.csv("L.csv")

str(mydata)

# Prepare lung data for survival analysis

lung <- survival::lung %>% mutate(status = as.numeric(status))

# Create a survival analysis task

task <- TaskSurv$new(id = "lung", backend = lung, time = "time", event = "status")

# Handle missing data with decision tree (for continuous variables)

task$missings()

# Use regression tree (rpart) for imputation

po <- po("imputelearner", lrn("regr.rpart"))

task <- po$train(list(task))[[1]]

task$missings()

# Extract imputed data

imputed_data <- task$data()

# Set random seed for reproducibility

set.seed(12341234)

# Select and configure learner

learner <- lrn("surv.glmnet", alpha = 1)

# View learner's parameters

learner$param_set

# Define search space for tuning

search_space <- mlr3verse::ps(s = p_dbl(lower = 0.001, upper = 2))

# Set up grid search tuner

tuner <- tnr("grid_search", resolution = 10)

# Perform cross-validation and automatic tuning

k <- 5

at <- auto_tuner(

learner = learner,

resampling = rsmp("cv", folds = k),

measure = msr("surv.cindex"),

search_space = search_space,

tuner = tuner,

terminator = trm("none"))

# Start the tuning process

set.seed(12341234)

at$train(task)

# View tuning results

at$tuning_result

# Plot the effect of parameter changes on model performance

at$archive %>%

as.data.table() %>%

ggplot(aes(s, surv.cindex)) +

geom_line(color = "steelblue", size = 1) +

geom_point(color = "red", size = 4) +

scale_x_continuous(breaks = seq(0, 2, 0.2))

# Set optimal parameters and retrain model

learner$param_set$values <- at$tuning_result$learner_param_vals[[1]]

learner <- lrn("surv.glmnet", alpha = 1, s = at$tuning_result$s)

set.seed(12341234)

learner$train(task)

# Plot model coefficients

model <- learner$model

plot(model, xvar = "lambda", label = TRUE)

# Cross-validation with glmnet

learner_cv <- lrn("surv.cv_glmnet", alpha = 1, nfolds = 5)

learner_cv$param_set$values <- at$tuning_result$learner_param_vals[[1]]

set.seed(12341234)

learner_cv$train(task)

# Display the cross-validation model

learner_cv$model

# Plot the cross-validation results

plot(learner_cv$model)

mlr3viz::autoplot(learner_cv$model)

# Extract and save optimal lambda value

lambdamin <- learner_cv$model[["lambda.min"]]

learner$param_set$values$lambda <- lambdamin

set.seed(12341234)

learner$train(task)

# Extract regularization coefficients and filter important variables

coef_matrix <- as.matrix(coef(model, s = at$tuning_result$s))

coef_df <- as.data.frame(coef_matrix) %>%

set_colnames(c("coef")) %>%

mutate(coef = as.numeric(coef)) %>%

filter(abs(coef) > 0) %>%

arrange(-coef)

# Save the selected variables and lambda value

lasso_gene <- rownames(coef_df)

save(lasso_gene, lambdamin, file = "lasso_gene.Rdata")

# Display selected variables

lasso_gene

Figures 3, 4, 5, and 6 were also created using the Storm statistical platform.

Figure 7 was generated using SPSS analysis.

Figure 8, the RCS plot, was created using R code:

rm(list=ls()) # Clear the current workspace

setwd("C:/Users/Dell/Desktop")

mydata <- read.csv("L.csv") # Import CSV data

View(mydata) # View data

install.packages("plotRCS")

library(plotRCS)

# Univariate RCS

library(rms)

library(Hmisc)

# Prepare the data

dd <- datadist(mydata)

options(datadist = 'dd')

# Build the regression model

mod <- lrm(status ~ rcs(Age, 4), data = mydata, x = TRUE, y = TRUE)

mod

# Check for linearity

an <- anova(mod)

# Calculate predicted values

OR <- Predict(mod, Age, fun = exp, ref.zero = TRUE)

OR

ggplot(OR)

# plot

library(ggplot2)

ggplot() +

geom_line(data = OR, aes(Age, yhat),

linetype = "solid", size = 1, alpha = 0.7, colour = "#0070b9") +

geom_ribbon(data = OR,

aes(Age, ymin = lower, ymax = upper),

alpha = 0.1, fill = "#0070b9") +

theme_classic() +

geom_hline(yintercept = 1, linetype = 2, size = 1) +

geom_vline(xintercept = 66.89559, size = 1, color = '#d40e8c') + # HR=1 corresponding to age

labs(x = "Age", y = "HR (95%CI)")

# Build the regression model

mod1 <- lrm(status ~ rcs(Age, 4) + Sex + Race + Mari + AJCC + Sums + Radiation + Chemotherapy + size + RNP + income + Surgery, data = mydata, x = TRUE, y = TRUE)

mod1

# Calculate predicted values

OR1 <- Predict(mod1, Age, fun = exp, ref.zero = TRUE)

OR1

ggplot(OR1)

ggplot() +

geom_line(data = OR1, aes(Age, yhat),

linetype = "solid", size = 1, alpha = 0.7, colour = "#0070b9") +

geom_ribbon(data = OR1,

aes(Age, ymin = lower, ymax = upper),

alpha = 0.1, fill = "#0070b9") +

theme_classic() +

geom_hline(yintercept = 1, linetype = 2, size = 1) +

geom_vline(xintercept = 23.42535, size = 1, color = '#d40e8c') + # HR=1 corresponding to BMI

labs(x = "Age", y = "OR (95%CI)")
